# A Giant Aggressive Angiomyxoma of the Pelvis Misdiagnosed as Incarcerated Femoral Hernia: A Case Report and Review of the Literature

**DOI:** 10.1155/2016/9256749

**Published:** 2016-05-05

**Authors:** Alper Sozutek, Oktay Irkorucu, Enver Reyhan, Kemal Yener, Ali Ayberk Besen, Kivilcim Eren Erdogan, Gulfiliz Gonlusen, Figen Doran

**Affiliations:** ^1^Adana Numune Training and Research Hospital, Department of General Surgery, Division of Gastroenterological Surgery, Adana, Turkey; ^2^Adana Numune Training and Research Hospital, Department of Medical Oncology, Adana, Turkey; ^3^Department of Pathology, Cukurova University Medical Faculty, Adana, Turkey

## Abstract

Aggressive angiomyxoma (AA) is an uncommon mesenchymal tumor that is mostly derived from the female pelvic and perineal regions. AA is a locally infiltrative slow growing tumor with a marked tendency to local recurrence. Painless swelling located around the genitofemoral region is the common symptom; thus, it is often misdiagnosed as a gynecological malignancy or a groin hernia. A 35-year-old female patient who previously underwent surgery for left femoral hernia operation resulting in surgical failure was reoperated for a giant AA located in the pelvis. The tumor was completely excised with free margins. Histopathologic examination revealed an AA. The tumor size was measured as 24 × 12 × 6 cm with a weight of 4.2 kg. Immunohistochemically, the cells show positive staining with vimentin, desmin, estrogen, and progesterone receptor. S100, MUC4, CD34, and SMA were negative in the tumor cells. AA should be considered in the differential diagnosis of any painless swelling located in the genitofemoral region, particularly in women of reproductive age. The principle treatment should be complete surgical excision with tumor-free margins. Long-term follow-up and careful monitoring are essential due to its high tendency of local recurrence in spite of wide excision of the tumor. Adjuvant antihormonal therapy yields promising results for preventing recurrence.

## 1. Introduction

Aggressive angiomyxoma (AA) is an uncommon mesenchymal tumor which is predominantly encountered among adult females in reproductive age [1]. The tumor usually arises from the pelvic and perineal regions; however, uncommon localization has been reported in the literature [2, 3]. AA is a locally infiltrative slow growing tumor with a marked tendency to local recurrence. Although it is previously regarded as a nonmetastasizing tumor, its metastatic potential has been revealed in a few recent reports [4, 5]. Macroscopically, AA has a gelatinous appearance, and it is microscopically characterized by a myxoid stroma and abundant thin-thick walled vascular channels [1, 6]. The tumor is distinguished from other lesions by these histopathologic features. The most common clinical symptom is painless swelling at vulva or groin area. Because of this clinical manifestation, AA is often initially misdiagnosed as a gynecological malignancy or a groin hernia that leads to unnecessary surgical interventions [7, 8]. Complete surgical excision with tumor-free margins is still accepted as the main treatment method for AA [1–9]. Unfortunately, the highest recurrence rates after resection still remain a major surgical problem that should be solved.

Since AA was first described in 1983 by Steeper and Rosai [10], approximately 200 cases have been reported in medical literature up to date. Because of its rarity, the clinical presentation and the treatment method of the tumor have been described mostly based on individual case presentations. It is noteworthy that there is still a lack of knowledge about the clinical presentation, the management options, and the follow-up results of AA in the current literature. According to our view, reporting case series of these tumors may lead to a better understanding of how AA behaves. Hence, we aimed to contribute our case to the literature by presenting the surgical outcome of a patient who underwent radical surgery for a giant AA.

## 2. Case Presentation

**Figure 1:**
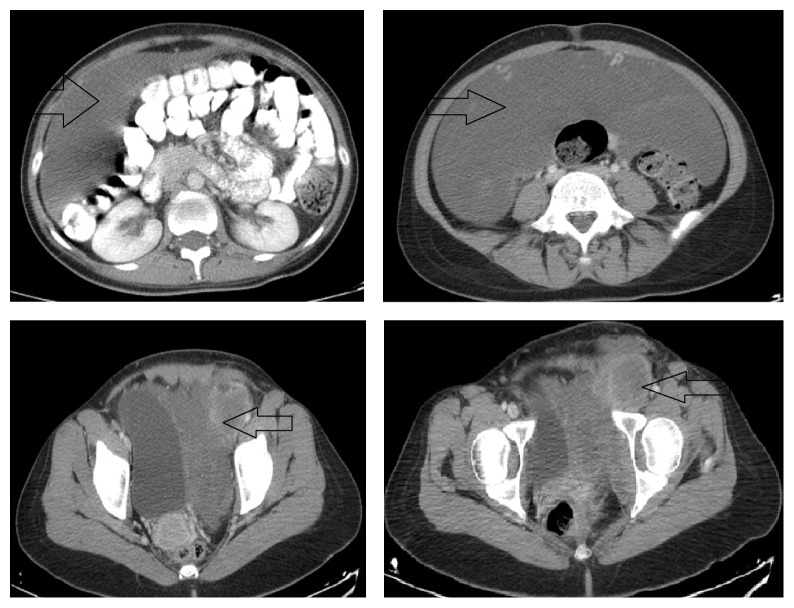
Computed tomography findings of the tumor.

**Figure 2:**
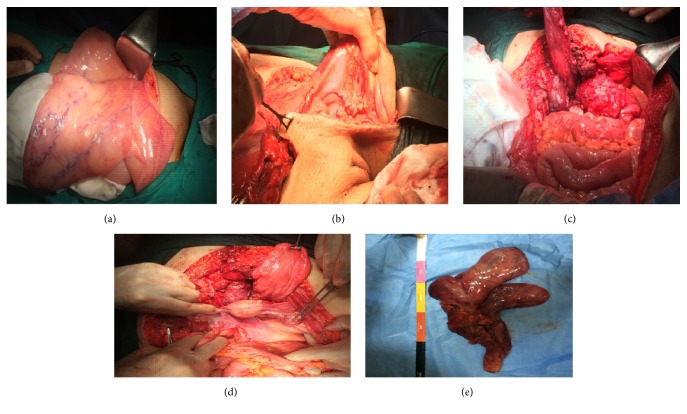
Operative findings of the tumor. (a) Macroscopic view of the tumor. (b) Extension of the tumor to the left thigh. (c) The radix of the tumor. (d) Abdominal view after accomplishing the surgery. (e) Resected specimen.

**Figure 3:**
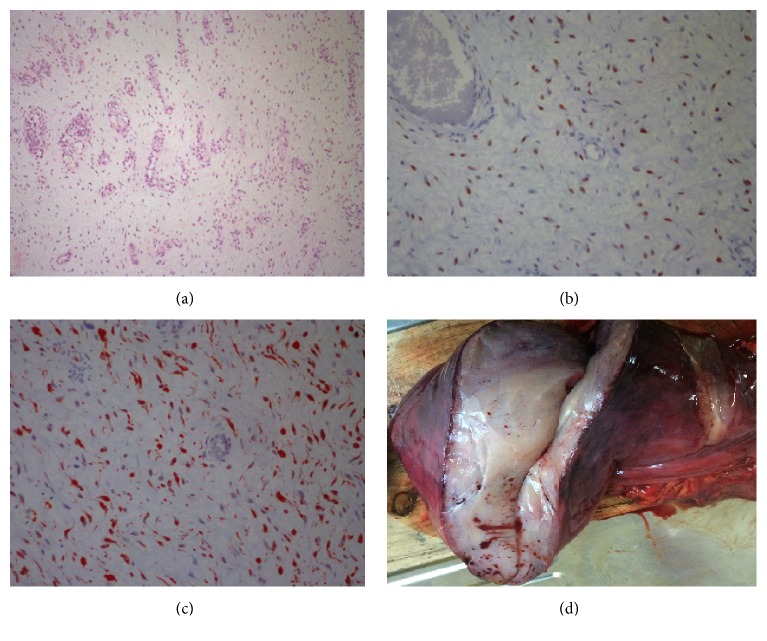
Histopathologic examination of the tumor. (a) Spindled and stellate-shaped cells in a myxoid and richly vascular background (H&E ×100). (b) Estrogen receptor immunoreactivity in aggressive angiomyxoma (H&E ×400). (c) Diffuse desmin immunoreactivity in aggressive angiomyxoma (H&E ×400). (d) Macroscopic imaging of the tumor.

A 35-year-old nulliparous female patient with a previous history of left femoral hernia operation was admitted to our hospital. In her medical history, the patient declared that she had a large painless swelling extending from her left groin to her left leg which grew gradually during one year. No gastrointestinal symptoms were determined. She was initially diagnosed as a femoral hernia and underwent surgery one month ago at another state hospital. Accordingly, she was referred to our clinic because of surgical failure. On physical examination, she had a left groin incision scar. An immobile, painless mass which filled lower quadrants of the abdomen was palpated. The mass was also extended to the one-third upper level of left thigh. Digital examination of the rectum was normal. Vulva was examined macroscopically because the patient was virgin. Subsequently, abdominal ultrasonography (USG), contrast-enhanced abdominal tomography (CT), and magnetic resonance imaging (MRI) were performed for estimating the tumor size, invasion degree of the mass, distant metastasis, and also ruling out other intra-abdominal lesions. A giant intra-abdominal solid mass that is fully filling the pelvis and extending to the left groin was revealed by USG, CT, and MRI (Figure 1). All adjacent organs and pelvic vessels were depressed by the mass. The origin of the tumor was not well demonstrated due to the huge size. Exploratory laparotomy was planned. A written informed consent including surgical risks was obtained from the patient. Laparotomy was performed through a midline incision that extends to the left thigh. On exploration, a giant, soft, rubbery, and gelatinous appearing mass (like lung tissue) that approximately filled the whole abdomen was noted. The tumor has exerted pressure on the bladder, the left ureter, uterus, and left iliac vein. The bladder and uterus were completely displaced to the right side. The tumor was extending to the left thigh via canal of Nuck. During the dissection, no infiltration into the adjacent organs was detected except a partial invasion into the left external iliac vein. This side was resected and primarily repaired. The external iliac artery was intact. The radix of the tumor was reaching until the posterior of the vagina. The tumor was completely excised by sharp and blunt dissection (Figure 2). The tumor size was measured as 24 × 12 × 6 cm with a weight of 4.2 kg. On histopathologic examination, the border of the tumor was clear. Morphologically, the tumor was composed of spindled and stellate-shaped cells with ill-defined cytoplasm intermingled with collagen fibers and thin vessels in a myxoid background. The cells had small round hyperchromatic nuclei with small centrally located nucleoli. At the periphery of the lesion, the vessels were thicker due to perivascular hyalinization and medial hypertrophy. The tumor was invading adjacent soft tissue including adipose tissue, muscles, and nerves. The mitotic activity was not observed. Immunohistochemically, the cells show positive staining with vimentin, desmin, estrogen, and progesterone receptor. S100, MUC4, CD34, and SMA were negative in the tumor cells (Figure 3). According to the histopathological and immunohistochemical findings, the case was interpreted as intra-abdominal aggressive angiomyxoma. The patient recovered uneventfully and was discharged from the hospital on postoperative day 8. The patient displays no evidence of local recurrence for 2 years postoperatively.

## 3. Discussion

AA is an uncommon mesenchymal tumor which is mostly derived from the pelvic and perineal regions including vulva, vagina, bladder, and rectum [1, 6, 8]. However, uncommon localizations such as lung, liver, larynx, and orbit have been reported [2, 3, 11–13]. It is hard to define the exact incidence of AA among the other intra-abdominal mesenchymal tumors because of its rarity. Although it is almost exclusively encountered among females in reproductive age, rare cases have been diagnosed in the perimenopausal female, children, and male patients [7]. The female-to-male ratio has been reported as 6.6/1 [14]. The reported age of presentation ranges from 1 to 82 years, with a median age of 33 years [6, 7]. In view of these data, the demographic characteristic and the tumor localization of our patient were similar to the majority of previously reported cases.

The pathogenesis of AA is not well understood. There is only one study evaluating the pathogenesis of AA in the current literature in which Nucci and Fletcher [15] suggested a translocation at the level of chromosome 12 where the high mobility group protein HMGA2 (a transcription factor expressed during embryogenesis) is located. AA is regarded as an aggressive tumor due to the neoplastic nature of the blood vessels and its high tendency of local infiltration and local recurrence. AA is considered as an invariably benign tumor; however, in a few recent reports, its metastasis to lungs resulting in death has been revealed. This condition which highlights the need to consider AA may be potentially malignant in some cases [4]. AA should be distinguished from other relatively more common encountered benign or malign soft-tissue sarcomas of the abdomen such as myxoma, fibrous histiocytoma, angiofibroma, liposarcoma, nerve sheath tumor, mixed mesodermal tumor, and angiomyofibroblastoma (AMF) [1, 2]. AA is distinguished from the other lesions by its immunohistological findings. AA is derived from myofibroblasts as a phenotypic variant of the basic fibroblast with a prominent vascular component [6]. The origin of the tumor may act like a wound healing situation and this may be the reason of its locally invasive character. Immunohistochemical staining of the tumor reveals high positivity for desmin, vimentin, ER, and PR receptor; however it generally reveals negativity for S-100 protein [1, 2, 6, 16]. These findings usually help us to distinguish from other mesodermal origin tumors. According to the immunohistochemical findings of the present tumor, our case was diagnosed as AA.

The diagnosis of AA is very difficult because it is often asymptomatic until the tumor reaches large sizes. Urinary, gynecologic, and gastrointestinal symptoms like dysuria, dysmenorrhoea, constipation, and chronic abdominal/pelvic pain occur when the tumor begins to depress the adjacent organs including bladder, rectum, ureter, and uterus. AA commonly manifests a painless swelling located around the genitofemoral region. For this reason, it is often misdiagnosed as a vulvar abscess, Bartholin's gland cyst, vaginal prolapse, gynecologic malignancy, and groin/femoral hernia that may lead to unnecessary surgical interventions, as in our case [9, 17]. In the present patient, AA was misdiagnosed as incarcerated femoral hernia and the patient underwent emergent surgery which resulted in surgical failure. Interestingly, preliminary diagnosis was not previously confirmed by any additional imaging studies such as USG, CT, or MRI. It is notable that preoperative imaging has a crucial importance in the diagnosis of AA. USG reveals hypoechoic cystic mass and usually remains insufficient for diagnosis. CT reveals a well-defined, hypoattenuated enhanced mass with a swirling appearance which is detected in about 83% of the patients [8, 18]. MRI is more helpful than the other imaging studies. On T1-weighted MR imaging, the tumor shows isosignal compared to the muscles while on T2 high signal intensity is detected. It is likely due to the loose myxoid matrix and high-water content of AA [8]. Furthermore, because the size of the tumor is often underestimated by clinical examination, these imaging studies also help us in deciding the surgical strategy. In view of these findings, we think that there is still a lack of knowledge about the diagnosis of AA among the clinicians. Thus, we emphasize that although it is a rare condition, particularly, a female in reproductive age with a painless swelling located around the genitofemoral region should be well examined by imaging studies to rule out AA and to decide the surgical strategy, as well.

The current treatment of AA is complete surgical excision with tumor-free margins [1, 6, 8]. However, there is still a debate about the treatment because of high recurrence rates in spite of wide surgical excision. The recurrence rate is reported with a wide range from 33% to 83% [10, 19]. Recurrence has been developed mostly within the first 3 years [14]. However, it may be detected even after postoperative 15 years [11]. Our patient displays no evidence of local recurrence for 2 years postoperatively. In a retrospective review by Chan et al. [14] and Han-Geurts et al. [20], it has been suggested that patients having positive margins were as likely to have recurrence as those with negative margins. Also, the tumor size is not correlated with recurrence. Thus, extensive surgery can be disregarded in patients with high morbidity and for preserving fertility, as well. Respecting this view, we believe that it is not compatible with the oncological principles. Incomplete or partial resection may lead to high recurrence rates. Furthermore, considering the recently reported cases with distant metastasis, complete resection should be performed as technical as possible despite the high morbidity of the operation. In the present case, surgery was performed after obtaining patient's informed consent including all surgical risks such as infertility, colostomy, and loss of organ. According to our view, partial resection should only be considered in cases refusing these surgical risks or in the presence of unresectable tumor.

All adjuvant treatment modalities remain controversial [8]. Chemotherapy yields no beneficial results for adjuvant therapy because of low mitotic activity of the tumor. Embolization of the tumor has been reported as an alternative approach; however, it remains insufficient due to the extensive vascular network of the tumor. The main localization of AA, which is limited to reproductive organ region, and the positive ER and PR status of the tumor suggest that AA may be a hormone-responsive neoplasm [1]. Several beneficial results with tamoxifen or gonadotropin-releasing hormone (GnRH) agonist have been described [1, 9, 17]. Fine et al. [21] achieved a complete resolution of a recurrent AA in a female patient who refuses redo surgery. However, long-term use of these drugs is associated with side effects such as menopausal symptoms and bone loss. Moreover, the optimal duration of therapy is unknown. The immunohistochemical findings of the present tumor confirmed positivity for both estrogen and progesterone receptors. Our patient received tamoxifen, 20 mg orally per day for 6 months, since we share the same view with Nakamura et al. [1]. We also suppose the use of antihormonal therapy as an adjuvant therapy for AA to prevent recurrences like in breast cancer.

Although the majority of the authors have reported no advantage in using radiotherapy, it can be a good alternative treatment in patients who are resistant to antihormonal therapy, those with recurrence or in whom tumor resection would cause high morbidity. Rhomberg et al. [22] and Suleiman et al. [23] have achieved local control by radiotherapy in patients with local recurrence.

## 4. Conclusion

Despite its rarity, AA should be considered in the differential diagnosis of any painless swelling located in the genitofemoral region, particularly in women of reproductive age. The diagnosis should be confirmed by CT and/or MRI. The principle treatment should be complete surgical excision with tumor-free margins. The patient should be informed about the high morbidity of the surgical intervention. Long-term follow-up and careful monitoring are essential due to its high tendency of local recurrence in spite of wide excision of the tumor. Adjuvant antihormonal therapy yields promising results for preventing recurrence. However, long-term use of these drugs is still controversial because of their adverse effects.
